# Cardiopulmonary and inflammatory biomarkers in heartworm disease

**DOI:** 10.1186/s13071-017-2448-2

**Published:** 2017-11-09

**Authors:** Elena Carretón, Rodrigo Morchón, José Alberto Montoya-Alonso

**Affiliations:** 1 Faculty of Veterinary Medicine, Research Institute of Biomedical and Health Sciences (IUIBS), University of Las Palmas de Gran Canaria, Trasmontaña s/n, 35413-Arucas, Las Palmas, Spain; 2 Laboratory of Parasitology, Faculty of Pharmacy, Institute of Biomedical Research of Salamanca(IBSAL), University of Salamanca, Salamanca, Spain

**Keywords:** Heartworm, Biomarkers, Troponins, Myoglobin, Acute phase, Proteins, C-reactive protein, D-dimer, NT-proBNP, Endothelin-1, Haptoglobin

## Abstract

In heartworm disease, several biomarkers of cardiopulmonary injury and inflammatory activity have been studied during the recent years. D-dimer is a fibrin degradation product present after a clot is degraded, which has been reported to provide support for the diagnosis of pulmonary thromboembolism in heartworm disease. Furthermore, concentrations increment with increased disease severity and during the adulticide treatment. This increase in concentration has proved to be valuable. Cardiac biomarkers troponin I, myoglobin and NT-proBNP demonstrated presence of myocardial injury and heart failure, especially in chronic infections, which in some cases, slightly improve after the adulticide treatment. An acute phase response in dogs with *Dirofilaria immitis*, characterized by variations of acute phase proteins (APP), has been reported, indicating inflammatory processes that could contribute to disease progression. Among them, C-reactive protein (CRP) increases according to the severity of the disease; and a strong correlation between pulmonary hypertension and CRP has been observed. In cats, little work has been done to ascertain the utility of these biomarkers in feline heartworm; the only published study in *D. immitis–*seropositive cats reported significantly higher concentrations in positive APP serum amyloid A, haptoglobin and ceruloplasmin.

## Background

### Canine heartworm

Heartworm disease is a vector-borne disease caused by the nematode *Dirofilaria immitis*. The infection is mainly characterized by the presence of adult worms in the pulmonary arteries. Furthermore, the existence of microfilariae and the release of the endosymbiotic bacteria *Wolbachia pipientis* also contribute to the pathophysiologic response to the infection [1–3].

**Fig. 1:**
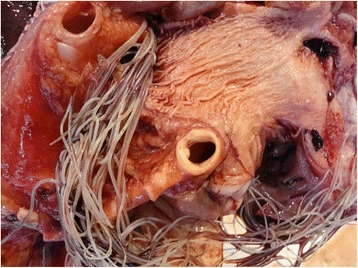
Macroscopic view of the proliferative endarteritis in the main pulmonary artery caused by chronic infection with heartworm disease in a dog that lived in a hyperendemic area of *D. immitis*

In dogs, the primary damage is caused to the pulmonary arteries and lung through the adult worms living in the pulmonary arteries, this damage starting very soon after the arrival of the worms. The changes manifest as vascular inflammation, endothelial damage and sloughing, villous proliferation of the intima, and the activation and attraction of leukocytes and platelets, which release factors that induce smooth muscle cell proliferation with collagen accumulation and fibrosis. The developing proliferative endarteritis causes occlusion of the vascular lumen; and the arteries become enlarged, thick-walled, tortuous and stiffer (Fig. 1) [2–4].

Moreover, worms that have died naturally or have been killed by adulticide treatments can produce pulmonary thromboembolism, granulomatous inflammation, arterial obstruction, and vasoconstriction. Furthermore, hypoxia caused by ventilation-perfusion imbalance secondary to pulmonary thromboembolism and release of vasoactive substances by vascular endothelial cells results in a prolonged vasoconstrictive status. This entire situation leads towards a hypertensive state and, as consequences, development of pulmonary hypertension and increased afterload of the right ventricle, which can produce right-sided congestive heart failure [2–4].

Several studies have aimed to assess the utility of the cardiopulmonary and inflammatory biomarkers in this matter. This has occurred in the context of awareness of the difficulties involved in the detection of the pathological alterations characteristic of the disease as well as the need to find new tools to help to determine the clinical status of the heartworm-infected dog, stage the disease and establish an accurate prognosis. The main purpose of this review is to gather and describe the current knowledge about cardiopulmonary and inflammatory biomarkers in canine and feline heartworm.

### History and utility of cardiac biomarkers

The use of cardiac biomarkers is quite recent. The first use was published in 1954, when aspartate aminotransferase (AST) was measured to assess the myocardial damage in a group of human patients as an aid in the diagnosis of acute myocardial infarction [5, 6], followed by lactate dehydrogenase (LDH) a year later [7] and joined by other biomarkers in the following years. These first biomarkers did not show cardiac specificity and presented low sensitivities. Then 1975 saw the beginning of assays for the detection of monoclonal antibodies with the development of serum myoglobin to detect presence of myocardial infarction [8, 9]. This was a quantum leap in the field of clinical diagnosis; a new generation of biomarkers based on the detection of antibodies was born. In the 1980s, immunoassays for the detection of cardiac troponins in myocardial infarction were developed [10, 11]. Since then, the number of biomarkers has increased notably, and today biomarkers are at the forefront of the diagnosis and monitoring of cardiac diseases.

The term *biomarker* was first used in 1989 [12], although it was not until 2001 that the definition was established as “a characteristic that is objectively measured and evaluated as an indicator of normal biological processes, pathogenic processes or pharmacologic responses to a therapeutic intervention” [13].

Encouraged by the positive results found in human cardiology, the use of biomarkers for the detection of cardiovascular diseases in veterinary medicine has become highly attractive and has been increasingly studied over the past 18 years [14]. Several human immunoassays have demonstrated an adequate sensitivity for the diagnosis of cardiac diseases in veterinary medicine, in addition to the growing development of commercial tests specifically for small animals. So far, dozens of studies have been published evaluating the cardiac biomarkers. In particular, they allow the detection of cardiopulmonary diseases in early or asymptomatic stages, confirm doubtful cases and processes difficult to identify, and can be used as tools to monitor a disease or to establish a prognosis and decide a specific treatment [14–17].

### Biomarkers of myocardial integrity in canine heartworm

Troponins are structural proteins, part of the contractile apparatus of skeletal and cardiac muscle tissue where they are responsible for regulating the interaction of actin and myosin in the control of muscle cell contraction. Troponin is composed of three subunits: troponin C, the calcium binding subunit; troponin I, the inhibitory component; and troponin T, the tropomyosin-binding subunit. In the cardiomyocytes, cardiac troponin I and troponin T are encoded by genes distinct from those encoding the skeletal muscle isoforms which are, thus, specific to the myocardial muscle [18].

As the troponins are purely intracellular proteins, their presence in circulation reflects intracellular content release from cardiomyocytes [19]. Most of the troponin in the cell is structurally bound in the contractile apparatus, while a small amount is free cytosolic troponin. When destruction of a cardiomyocyte occurs, there is a quick release of the cytosolic pool with an early rise in circulating troponin, followed by the slower release of the structural pool as the contractile apparatus is broken down, resulting in a sustained increase in circulating troponin for days to weeks [20–22]. Also, if the damage is minor, the cytosolic pool alone may also be released independently [21, 23]. Therefore, circulating concentrations of troponins provide information about cardiac-specific injury and, thus, are biomarkers that evaluate the myocardial integrity. Elevated levels are detectable in the blood within 4 h, reach a peak within 12 to 24 h and then slowly decline over the next 5–20 days, depending on the severity of damage [10, 11, 24, 25]. There are numerous studies that demonstrate the utility of both troponin T and troponin I in the evaluation of myocardial damage in different pathologies in small animals, as the slightest increase in serum levels indicates presence of myocardial injury [16, 17, 26, 27].

Cardiac troponins have been evaluated in dogs with dirofilariosis, finding that troponin T levels were normal in dogs with heartworm; but troponin I levels were significantly higher than those of healthy seronegative dogs [28, 29]. Furthermore, it was reported that dogs with heartworm and higher levels of troponin I correlated with vertebral heart size scores greater than the normal range and existence of ECG alterations in 66% of the animals, which may be indicative of presence of myocardial injury [30]. Even slight rises of troponins are indicative of myocardial damage. Therefore, in view of the results of these studies, presence of myocardial injury in dogs with heartworm can be confirmed.

The reason why the levels of troponin T were normal was attributed to the difference in size of the molecules of troponin. Cardiac troponin T has a molecular weight of 37 kDa while cardiac troponin I is a slightly smaller protein of 24 kDa [31], so less severe cardiac damage allows leakage of this troponin to occur more easily. It has also been suggested that troponin T is more tightly bound to the contractile apparatus [32, 33]. For this reason, troponin I has been shown to increase more frequently and at an earlier stage of several small animal cardiac diseases, compared with troponin T [26, 27]. Accordingly, an increase of both troponin T and troponin I concentrations appears to reveal more severe cardiac xinjury. For this reason troponin I is considered a more sensitive and specific marker than troponin T for the detection of minor myocardial damage [16, 17, 26, 27].

Myoglobin is also used as a biomarker of myocardial integrity. It is a major protein of skeletal and cardiac muscle sarcoplasm of 17.8 kDa and functions as an oxygen store in oxidative fibers. Myoglobin is located in skeletal and cardiac muscle and represents 2% of the cytoplasmic protein in these cells, so it is not cardiospecific [34–36]. Its main advantage lies in its quick release after an ischemic event: in human patients with myocardial infarction, myoglobin levels rise within 2 h, peak between 6 and 9 h and return to normal within 24 to 36 h [34, 37]. This is why it has been a very useful tool in the early diagnosis of acute myocardial infarction in human medicine [37, 38].

In veterinary medicine, however, its utility is unclear; detection of elevated plasma myoglobin in dogs is not considered clinically useful due to its low specificity, rapid elimination and the low incidence of ischemic cardiac disease in dogs compared with humans. There are only a few publications that show elevated concentrations of myoglobin in dogs with cardiac damage in a range of pathologies, such as gastric dilatation-volvulus, blunt chest trauma, and systemic hypotension. The results provide interesting information despite not being able to confirm whether the origin of the elevations of myoglobin is cardiac or skeletal [39–42].

In heartworm, the studies showed elevated circulating myoglobin values in 20.8% to 26.6% of the dogs. Pathological presence of myoglobin was more frequent and concentrations were especially high in the microfilaremic dogs in all of the published studies [29, 30, 43]. Due to the nonspecificity of this protein, however, it was not possible to determine if the origin was cardiac or skeletal or what role microfilariae played in this increase.

**Fig. 2:**
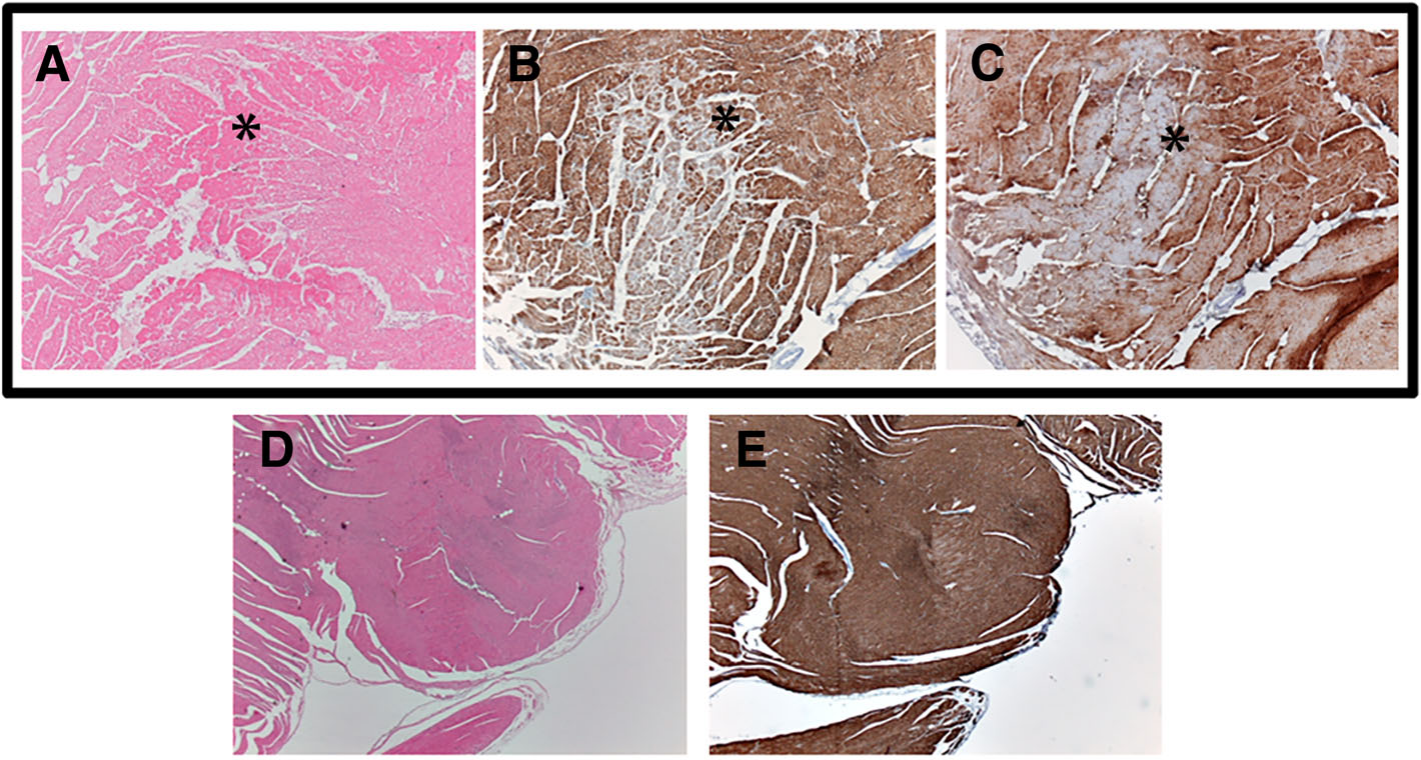
Histology and immunohistochemistry of right ventricle from two dogs with heartworm. Dog 1: (**a–c**) the asterisk points the presence of necrosis (**a**, H&E, 20×), which corresponded in the same area with a decreased staining for troponin I (**b**, immunostaining for troponin I, 20×) and myoglobin (**c**, immunostaining for myoglobin, 20×). (**d–e**) healthy myocardial tissue (**d**, H&E, 10×) and immunostaining of the same area where a consistent and uniform staining was observed (**e**, immunostaining for troponin I, 10×). Images courtesy of the coauthors of Carreton et al., Reference [43]

The myocardial origin of the increased plasma levels of these two proteins was confirmed by an immunological study that examined by histology and immunohistochemistry the right ventricles of 24 infected dogs with antimyoglobin and troponin I antibodies [43]. In these samples, the main histologic feature in myocardial tissue was focal necrosis; frequent patches of hypereosinophilic, necrotic myocardium were also described as well as occasional neutrophilic inflammatory infiltrates (Fig. 2). Where necrosis was present, there was a consistent loss of staining for myoglobin and troponin I, indicating increased concentrations of myoglobin and troponin originated by myocardial damage in these dogs [43].

The utility of these biomarkers was also evaluated in order to assess the severity of the disease [44]. In one study, 20 heartworm-infected dogs were divided into four groups based on the severity of the disease, according to the classification established by Di Sacco and Vezzoni [45]. In this study, dogs from classes I and II showed normal concentrations of troponin I and myoglobin. Troponin I was slightly increased in class III, while 40% of dogs had abnormally elevated concentrations of myoglobin. Dogs from class IV had the highest levels of troponin I, with 60% of these elevated beyond reference values of myoglobin as well as having the highest average values [44]. It seemed that class I and II dogs did not suffer significant myocardial damage, and the injury to the myocardium became evident as the disease became chronic. Since the biomarkers of myocardial integrity progressively increase with worsening degrees of heartworm disease, their use could be helpful in the staging of the severity of the disease [44].

Once these biomarkers’ value and usefulness had been established, they were also evaluated during adulticide treatment [30]. After the pretreatment with doxycycline and ivermectin, the concentrations decreased in all dogs. It seems that the elimination of *Wolbachia* and reduction of microfilariae may lessen the vascular inflammation and, therefore, reduce the severity of the disease [1]. In the case of myoglobin, values were within the normal range in all dogs from this point and during the rest of the treatment. It was observed that troponin I levels decreased, reaching their lowest concentrations at the end of the treatment in dogs with high concentrations of this biomarker on the day of the diagnosis, although levels still remained above reference values. On the other hand, the dogs with low concentrations of troponin I on the day of diagnosis showed normal levels during all treatment, indicating that there is not significant myocardial damage following the recommended adulticide protocol [30]. As the pathology disappears, there is an improvement; but, if there is myocardial damage, this appears to persist. There are similar results described in human medicine in patients with chronic congestive heart failure compensated for by conventional treatment, in which there are sometimes persistent high values of troponin I [46]. It is also reported that, for the human patient, an increase of troponins over time could be associated with a higher risk of death and, conversely, that outcome tends to improve in patients with decreasing concentrations [47, 48]. This has been observed in dogs, in a study of several heart diseases that revealed a significantly higher risk of death with increasing concentrations of cardiac troponins [49]. In view of the results, further studies to evaluate evolution and quality of life of these dogs would be highly interesting.

In this study the higher troponin I concentrations corresponded with dogs that had a high parasite burden. There are other factors, however, that have not been not evaluated in the study, which also may cause elevations of troponin I, such as the presence of pulmonary hypertension [50], which may complicate being able to draw conclusions from these data.

### Natriuretic peptides

To evaluate the presence of heart failure, other biomarkers have been evaluated in heartworm: N-terminal pro B-type natriuretic peptide (NT-proBNP) and atrial natriuretic peptide (ANP). B-type natriuretic peptide is primarily produced by ventricular myocytes, although the atria also contribute a small amount to their synthesis [51]. B-type natriuretic peptide is released as prohormone into circulation in response to other neurohormonal peptides and to a variety of stimuli, such as volume overload, cardiac hypertrophy and hypoxia. During its release, the prohormone is cleaved enzymatically into two fragments: the biologically active form BNP, which leads to natriuresis and vasodilation, and the inactive fragment NT-proBNP. B-type natriuretic peptide and NT-proBNP are metabolized via separate pathways, resulting in a difference of half-life between the two fragments. In dogs, the half-life of BNP is approximately 90 s [52], whereas the half-life of NT-proBNP is unknown, although in humans is approximately 120 min [53].

The utility of this biomarker has been positively evaluated in several cardiac diseases in small animals. It has been proven useful to confirm the diagnosis of a cardiac disease, to evaluate the efficacy of a treatment and to assess the severity of the disease; and it has been demonstrated that higher concentrations of these biomarkers correlated with lower median survival, so presenting a prognostic value to predict survival [15, 54–57].

Only a single study so far has evaluated this biomarker in heartworm disease [44]. In that study 20 dogs were divided into 4 groups (*n* = 5 per group) based on severity of the disease [45]. Class I and II dogs presented normal values of NT-proBNP, while class III dogs showed pathological values and NT-proBNP values and in class IV were above the detection limits of the equipment [44]. These results are consistent with those from other studies involving biomarkers of myocardial integrity: cardiac damage is only observed in chronically infected dogs. In heartworm disease, the severity of the proliferative endarteritis is directly related to the duration of infection and worm burden [58, 59]; as the disease becomes chronic, the reduction of compliance of the pulmonary arteries and vasconstrictive status lead to a hypertensive pulmonary state and, as a consequence, an increased afterload of the right ventricle and right-sided congestive heart failure [2, 4]. It is also known that a decrease in extracellular collagen matrix occurs in the myocardium of heartworm-infected dogs, which may contribute to ventricular dilatation [60].

Other authors found a correlation between plasma levels of ANP, another natriuretic peptide released mainly from the atrium in response to atrial pressure, and pulmonary arterial pressures in dogs with heartworm [61, 62]. However, as NT-proBNP is also high in dogs with pulmonary hypertension, with or without congestive heart failure [63, 64], further research is necessary to assess the utility of these biomarkers either as cardiac biomarkers or markers of pulmonary hypertension. Although several biomarkers have been shown to increase in dogs with pulmonary hypertension, there does not appear to be a single test that can differentiate pulmonary hypertension from congestive heart failure at this time [16].

### D-dimer as biomarker of pulmonary thromboembolism

D-dimer is a final product of degradation of fibrin and is specific for the breakdown of insoluble cross-linked fibrin from clots that have formed. The term *D-dimer* refers to the fact that the fragment consists of two cross-linked D fragments of fibrin. Unlike fibrin(ogen) degradation products, D-dimer is only present when plasmin lyses the completely cross-linked fibrin of a fully formed clot and, therefore, should not be present in significant concentrations in a normal and healthy animal [65]. For this reason, the measurement of D-dimer is a more sensitive and specific test compared to testing for fibrin(ogen) degradation products for the laboratory diagnosis of disseminated intravascular coagulation (DIC) and thromboembolic disease in human [66, 67] and canine [68–71] medicine. D-dimer appears 1 h after the episode and remains increased for 7 days [72]. Of the available laboratory markers, only D-dimer has shown clinical utility in detecting early embolism in humans [66, 73, 74]. D-dimer is not specific to pulmonary thromboembolism but occurs when there is breakdown of any clot [75]. In veterinary medicine, diseases other than pulmonary thromboembolism, such as sepsis, heart failure, hepatic failure or neoplasia, have also been shown to have increased D-dimer [69, 76–78]. Physiologic clots formed following surgery or trauma can cause elevation of D-dimer as well [69, 79].

The difficulty in diagnosing pulmonary thromboembolism (PTE) in small animals may be compounded by its subtle symptomatic presentation, as well as a lack of clinical suspicion and noninvasive tests that are sensitive and specific to the diagnosis of PTE [69, 80]. Early recognition of this condition in critically ill dogs followed by instigation of antithrombotic therapy may help to reduce mortality in these patients [81]. Because increased plasma D-dimer concentration can be useful in the identification of dogs with thromboembolic disease and DIC, it is the biomarker of choice [68–71, 82–84]. Furthermore, it has a great value in excluding thromboembolism when there is suspicion and the biomarker is negative, since undetectable levels of D-dimer make the diagnosis of thromboembolism highly unlikely [69, 71].

**Fig. 3:**
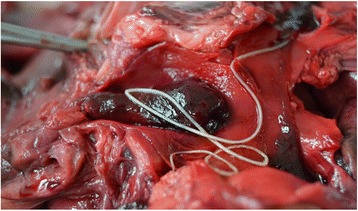
Thrombus in the left pulmonary artery of a dog with heartworm disease. This patient presented with high serum concentrations of D-dimer

The studies evaluating this biomarker showed that between 34.8% and 47% of dogs that tested positive for *D. immitis* antigen had D-dimer levels above the normal range, possibly due to the thromboembolic complications caused by the infection (Fig. 3) [29, 85, 86]. The presence of microfilaremia seemed to increase D-dimer values, suggesting that microfilariae could increase the tendency to develop thromboembolisms [29, 85]. Also, the parasite burden seemed to correlate with D-dimer concentrations; the highest mean values occurred in those dogs that had the highest parasite burdens [86].

**Fig. 4:**
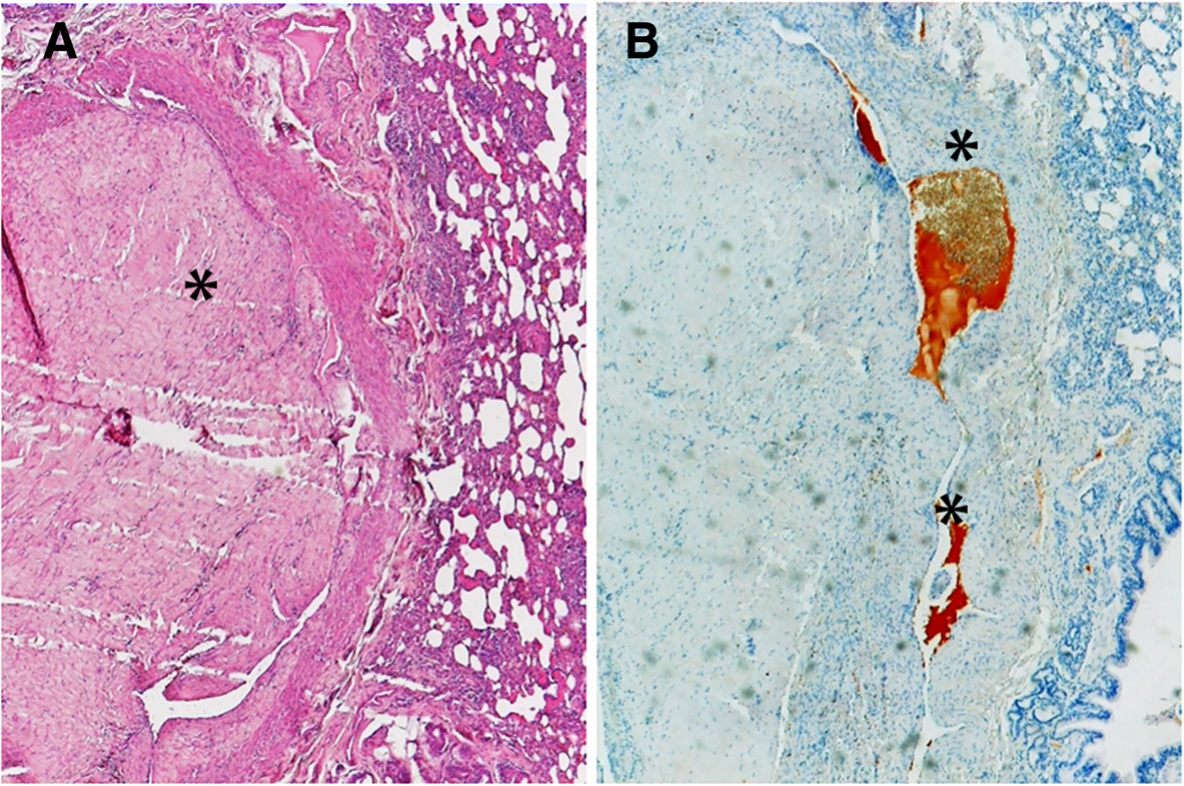
(**a**) pulmonary thromboembolism in lung tissue in a dog with heartworm (asterisk, H&E, 20×). (**b**) In the same area, presence of positive staining for D-dimer (asterisks, immunostaining for D-dimer, 20×). Images courtesy of the co-authors of Carreton et al. [85]

**Fig. 5:**
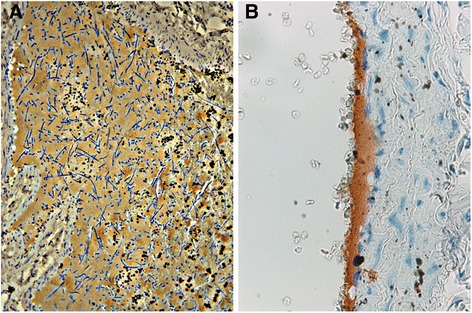
Immunostaining for D-dimer in pulmonary arteries of heartworm-infected dogs. **a** aggregates of microfilariae surrounded by positive staining for D-dimer (40×). **b** deposits of D-dimer in the endothelium of a pulmonary artery (100×). Images courtesy of the co-authors of Carreton et al., Reference [85]

To confirm the origin of the serum biomarker, a study evaluated the presence of D-dimer in lung and kidney tissue of infected dogs through immunohistochemistry [85]. In this study, positive staining for D-dimer was associated with the presence of PTE, often with worm fragments surrounded by clots (Fig. 4). Moreover, all dogs with pathological levels of plasma D-dimer were associated with positive staining for D-dimer in lung tissue. It was also noted that lungs of microfilaremic dogs were more frequently positive to D-dimer staining than amicrofilaremic dogs. In many cases it was observed that positive staining for D-dimer was associated with aggregates of microfilariae (Fig. 5). When the renal tissue of these dogs was evaluated, all microfilaremic dogs showed the presence of D-dimer within the glomerular capillaries while amicrofilaremic dogs were negative. This study concluded that the presence of microfilariae probably compromises renal function and contributes to renal pathology [85]. However, because the clearance of D-dimer is thought to be carried out by liver and kidneys, further research is necessary [87].

When the dogs were evaluated by groups based on severity of the disease, the following was noted [44]: in a study where 20 dogs were divided into 4 groups (*n* = 5 per group) according to classifications described by Di Sacco and Vezzoni [45], it was observed that D-dimer concentrations were within reference values in classes I and II, while the dogs showed abnormally elevated values in 40% of dogs from class III and 100% of dogs from class IV. Also, dogs from the former group presented the highest mean values [44]. The results presented evidences of thromboembolisms and/or DIC in class III and IV dogs and, no less important, absence in classes I and II, and demonstrated the utility that this biomarker may also have in the classification of the severity of the disease in dogs infected by *D. immitis*.

D-dimer was further evaluated during the adulticide treatment [86]. It was observed that, after the pretreatment with doxycycline and ivermectin, concentrations of D-dimer decreased, probably due to the elimination of *Wolbachia*, reduction of microfilariae and moderate exercise restriction, which may reduce the vascular inflammation and, therefore, reduce thromboembolic processes or its severity [1, 88], demonstrating the benefits of following specific adulticide guidelines aimed at limiting the pathology associated with dying worms [89].

All dogs with a high parasite burden showed elevated values of D-dimer on several occasions during the treatment, especially the first and the second weeks after the injections of melarsomine, which is surely due to embolization of the dead worms [86]. On the other hand, the dogs with low parasite burden showed elevated D-dimer levels during the treatment less often, and all of them showed undetectable levels of D-dimer from the third week after the first injection of melarsomine, which indicates that there are not significant thromboembolic processes from day 75 and probably most of worms die after the first injection of adulticide [86].

Dogs receiving prednisone presented high mean concentrations of this biomarker during the adulticide treatment [90]. Generally, prednisone is given to prevent and control adverse reactions of pulmonary thromboembolism in dogs that are more likely to have a significant worm burden; it is sometimes administered as routine prophylaxis [2, 4]. However, corticosteroid therapy could worsen the intimal disease, act as a procoagulant and also possibly reduce pulmonary blood flow when administered for several weeks [4, 91], therefore potentially exacerbating PTE. The increased risk of pulmonary embolism after a month of treatment with prednisone has also been demonstrated in human medicine [92, 93]. In light of this, the indications for routine use of corticosteroids in heartworm-infected dogs need to be critically reevaluated; and routine prophylactic administration is not recommended.

Following the recommended adulticide treatment [89], 1 month after the last injection of melarsomine, between 20% and 33.3% of the dogs with high parasite burden showed elevated concentrations of D-dimer, which indicated that thromboembolic processes were still occurring [86, 90]. Furthermore, when the concentrations of D-dimer were evaluated following the classic treatment (two injections of melarsomine administered 24 h apart without any pretreatment or adjunct therapy), 60% of dogs still presented elevated levels 1 month after the injections [90]. Therefore, the exercise restriction should continue in all of those dogs; and the assessment of the risk of PTE through the serial determination of D-dimer may be a useful tool to decide when the dog can safely return to normal activity levels after treatment.

**Fig. 6:**
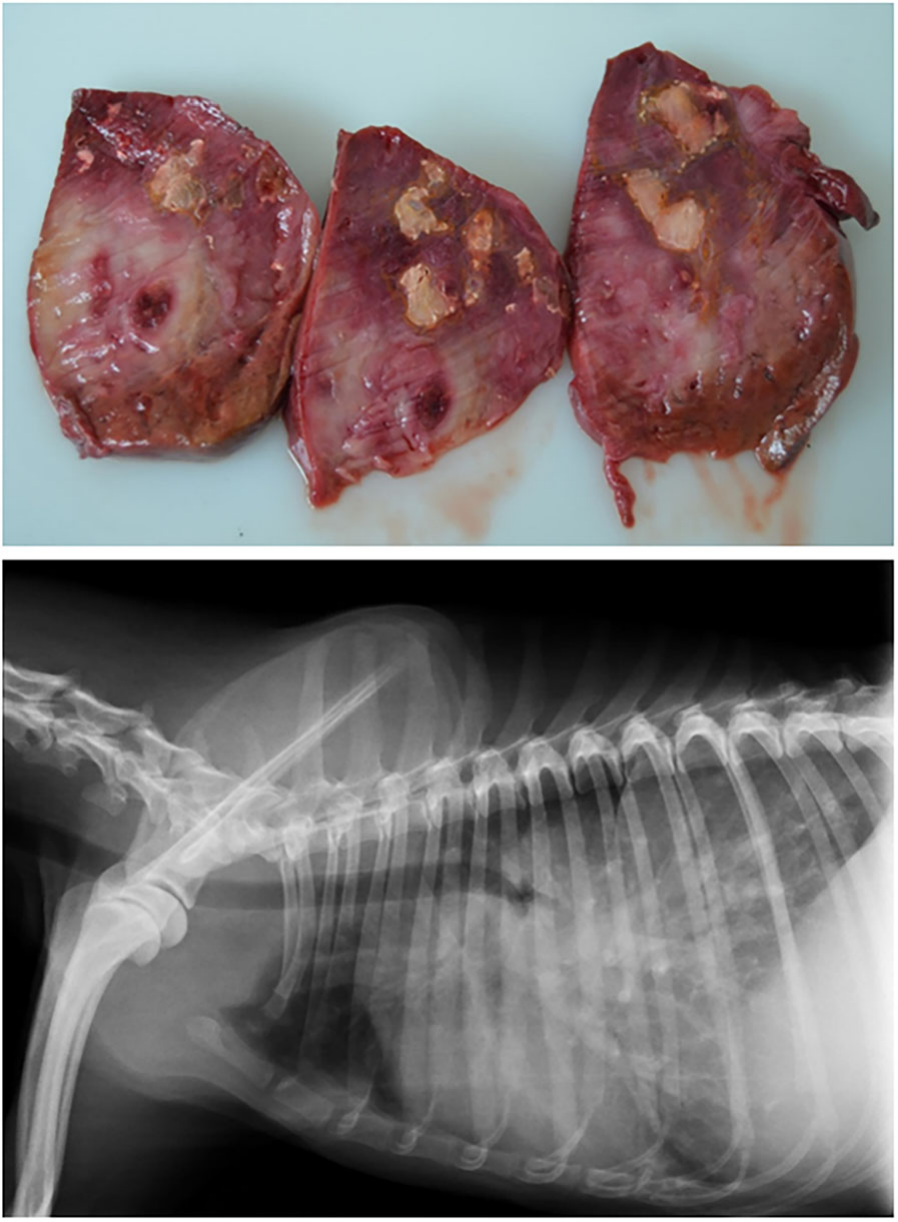
Macroscopic aspect of the lung of a dog that has chronic infection with heartworms. Thoracic radiographs were compatible with pulmonary thromboembolism. D-dimer analysis showed pathological concentrations. Despite efforts, the dog did not survive. Necropsy revealed presence of four to five adult parasites

The monitoring of D-dimer levels in infected dogs seems useful in evaluating the presence or absence of PTE. It also seems helpful to determine the severity of the disease, bearing in mind that clinical signs of PTE are highly variable, inconsistent and nonspecific, and many small animals with PTE will have normal thoracic radiographs [80]. Therefore, the diagnosis of this disorder is often difficult to obtain and is frequently missed. Furthermore, in most cases, the consequences of misdiagnosis are serious. Unfortunately, there is a lack of noninvasive tests that are sensitive and specific for the diagnosis of PTE. Although numerous laboratory biomarkers of coagulation have been studied, the D-dimer assay is considered the marker of choice for dogs with a suspicion of PTE [69]. Furthermore, negative D-dimer concentrations may exclude PTE in a suspicious patient. Because PTE is a frequent and serious condition in heartworm disease, the use of this biomarker could be extremely valuable (Fig. 6).

### Acute phase proteins

The acute phase response refers to a nonspecific and complex reaction in an animal that occurs shortly after any tissue injury. This response can originate from trauma, infection or inflammation with the purpose of restoring homeostasis and removing the cause of its disturbance [94–96]. The acute phase response is considered a part of the innate host defense system and is characterized by a number of different systemic effects, including fever, leukocytosis, increased blood cortisol, metabolic changes (ie, lipolysis, gluconeogenesis, muscle catabolism) and decreased serum iron and zinc concentrations [95]. During this response, there is a variation in the concentrations of certain proteins present in the plasma called acute phase proteins (APP). They are classified into two groups based on their response to the triggering event. Negative APP are those whose levels are diminished, while positive APP are those whose levels are increased when there is an acute phase response. Most positive APP are synthesized mainly by hepatocytes upon stimulation by proinflammatory cytokines (tumor necrosis factor alpha [TNF-α], interleukin-1 [IL-1] and interleukin-6 [IL-6]) and released into the bloodstream [95–97]. C-reactive protein (CRP) was the first described APP in 1930, originally named for its ability to bind the C-polysaccharide of *Pneumococcus* pneumonia [98].

The acute phase response is a very fast but highly nonspecific response because it develops secondary to numerous conditions that can produce tissue injury and occurs before the specific immune response. Although their physiologic role is still not well understood, it is apparent that APP are involved in regulation of the immune response, inflammation, protection against infection and in the repair and recovery of damaged tissue; the same individual APP can have pro- and anti-inflammatory effects, with a delicate balance between the two functions [94, 95, 97].

It has been published that the initial immune interactions between the host and the parasite are classified as innate immune response [99] and that there is an immune response against the adult worms, microfilariae and factors released by the parasites and *Wolbachia*, which induce the inflammatory response [1, 88]. Bearing that in mind, a few studies determined the concentrations of positive and negative APP in heartworm-infected dogs [100, 101]. The positive APP evaluated were CRP and haptoglobin, while the negative APP were albumin and paraoxonase-1 (PON-1). Dogs with dirofilaria displayed significantly higher concentrations of CRP and a significant decrease in albumin and PON-1 activity independently of the presence or absence of microfilariae, showing an acute-phase response that was probably due to the existence of vascular and pulmonary tissue damage associated with the presence of adult parasites, microfilariae and release of *Wolbachia* spp. [100, 101]. On the other hand, decreased concentrations of haptoglobin were observed, especially in microfilaremic dogs, representing a divergence between the behavior of the positive APP [100, 101]. This discrepancy was explained by the possible presence of hemolytic anemia, which could be clinical or subclinical; we can therefore speculate that the mechanism behind this would be that haemoglobin released from erythrocytes binds to and saturates haptoglobin and is removed from the circulation, as found in other conditions that produced intravascular hemolysis, such as canine babesiosis [102, 103].

CRP was also evaluated by groups of dogs based on the severity of the disease [44], according to classifications described by Di Sacco and Vezzoni [45]. The mean concentrations increased according to the severity of the disease so that class I dogs showed normal CRP concentrations, class II dogs showed a slight increase in the mean CRP concentrations, and classes III and IV presented pathological elevations of this APP, the mean concentrations of class IV being the highest [44]. This study revealed that the inflammatory response may be significant from the early stages of the infection, probably due to the role of *Wolbachia* spp. and the vascular damage caused by the adult worms in the pulmonary arteries, taking into consideration that the lesions begin immediately upon arrival of the parasites and worsen with the chronicity of the disease [4, 104].

Another study went a step further and found a correlation between CRP concentrations with severity of pulmonary arterial damage and pulmonary hypertension in dogs with *D. immitis* [105]. In this study, CRP was significantly increased in infected animals with mild or severe pulmonary hypertension. In humans, the pulmonary arterial damage that results from inflammation within the wall may create an increase in CRP concentration; and CRP is able to enhance smooth muscle proliferation and secretion of endothelin-1, a potent vasoconstrictor [106, 107]. Given the similarities to canine heartworm disease [2, 4, 104], it is possible that in a chronic process of remodeling of the arterial walls, the APP, especially CRP, may have a role and that their concentrations will be associated with severity and duration of disease. Therefore, there is potential for use of CRP for staging and monitoring disease in dogs and as an early biomarker of pulmonary hypertension for initial screening of dogs with heartworm disease [105]. This relationship between CRP and pulmonary hypertension was supported by the results of another study, which also evaluated haptoglobin, albumin and PON-1 [108], and confirmed the relationship between pulmonary hypertension and acute phase response, especially in the positive APP CRP and haptoglobin. These APP demonstrated a potential prognostic and diagnostic role, since increases correlated with presence and severity of pulmonary hypertension. Moreover, elevated concentrations persisted in dogs with pulmonary hypertension 1 month after the end of the adulticide treatment, while in dogs with normal pulmonary pressure levels they decreased or even returned to reference values [108]. The parasite burden seems to have no effect on CRP in the absence of pulmonary hypertension [105]. Mendez et al. reported higher concentrations of CRP in dogs with high parasite burden; however, the presence or absence of pulmonary hypertension was not assessed in this study [101].

A significant decrease in CRP concentrations after the treatment with doxycycline and ivermectin was observed [101], which probably reflected a reduction of the vascular inflammation caused by the elimination of the bacteria *Wolbachia*, demonstrating once again, the benefits of following the recommended adulticide protocols [88, 89]. CRP and haptoglobin increased considerably in the second week after the first injection of melarsomine, especially in dogs with high parasite burden, probably due to pulmonary inflammation and thromboembolism caused by the death of the worms [86].

### Endothelin-1

Endothelin-1 (ET-1) is a bioactive peptide produced by numerous cells, including vascular endothelial cells, vascular smooth muscle cells, airway epithelial cells, macrophages, fibrocytes, cardiac myocytes and others. It is a potent vasoconstrictor and promoter of cell proliferation, causing chronic structural changes in the cardiopulmonary tissues, leading to tissue remodeling [109–111]. In human medicine, ET-1 induces acute vasoconstriction and chronic vascular remodeling, which probably lead to the development of pulmonary hypertension [112–114]; and studies have demonstrated that ET-1 concentrations correlated strongly with the severity of pulmonary hypertension [113, 115]. A study reported that the plasma ET-1 levels in dogs with dirofilariosis were significantly increased, suggesting that ET-1 plays an important role in the pathophysiology of canine dirofilariosis as an aggravating factor by inducing pulmonary hypertension [116]. Expression of ET-1 is mainly induced by cytokines, angiotensin II, vasopressin, hypoxia and shear stress [117], which in dogs with heartworm could be triggered by an immunological reaction to the worms, hypoxia resulting from thromboembolisms and stress caused by the nematodes themselves. This theory was reinforced by other studies in dogs, which showed that ET-1 may have a role in the pathogenesis and disease progression in pulmonary hypertension [118, 119]. However, although ET-1 may be promising as an indicator of pulmonary hypertension, there are other pathological conditions that have been shown to cause ET-1 elevations, decreasing the specificity of this biomarker. Future studies should be carried out to determine its role in a clinical situation.

### Feline heartworm

Feline heartworm disease is a very different clinical entity from canine heartworm disease. Although recognized since 1921, the scientific community has only focused attention on this disease in the feline patient over the past 20 years. In the cat, the infection is basically pulmonary in nature; animals may be asymptomatic and, when present, signs are usually nonspecific, frequently respiratory or digestive. Sometimes, infected cats die suddenly without any premonitory signs [120–122]. The respiratory signs usually develop during the arrival and subsequent death of immature adult (juvenile) worms into the pulmonary arteries and during the death of the adult worms, attributed to an acute vascular and parenchymal inflammatory response caused by specialized pulmonary intravascular macrophages in the capillary beds of the feline lung whose activation is mainly responsible for the exacerbated pulmonary reaction [123, 124]. The result is an acute respiratory distress syndrome known as Heartworm-Associated Respiratory Disease (HARD) [125]. The presence of the bacteria *Wolbachia* has also been demonstrated as being involved in the development of the pulmonary inflammatory reactions in the feline host [126].

### Cardiopulmonary biomarkers

Apparently, D-dimer is not useful for the diagnosis of pulmonary thromboembolism in cats. There has been no research assessing the D-dimer levels in cats with pulmonary thromboembolism; however, they have been evaluated in cats with arterial thromboembolism, demonstrating that D-dimer concentration did not differ significantly from the median concentration in healthy cats; and only 50% of cats with arterial thromboembolism had increased D-dimer concentrations [127]. Regarding biomarkers of heart failure and myocardial integrity, no data have been published in cats with dirofilariosis to date.

### Inflammatory biomarkers

Only one study has been published so far on biomarkers in feline heartworm [128]. This study evaluated the positive APP haptoglobin, ceruloplasmin and serum amyloid A (SAA) in cats seropositive for anti–*D. immitis* and anti-*Wolbachia* antibodies. For that purpose, seropositive cats were divided into asymptomatic, symptomatic with clinical signs related to heartworm (mainly respiratory and digestive) and symptomatic with clinical signs triggered by other diseases. It was observed that SAA and ceruloplasmin concentrations were significantly higher in seropositive cats that showed compatible clinical signs when compared to seropositive and asymptomatic cats, or with clinical signs not compatible with heartworm disease. Also, haptoglobin was significantly higher in seropositive cats when compared with control animals. An association between feline seropositivity for *D. immitis* and an APP response was demonstrated, under the assumption that it was caused by the pulmonary inflammation induced by *D. immitis* and *Wolbachia*. The authors postulated that combining APP and seropositivity could increase the index of suspicion of heartworm as the cause of the clinical signs. In this study, however, the seropositivity of the feline patients only confirmed *D. immitis* exposure, and the cats were not necessarily infected; so to determine the utility of the APP in feline heartworm infection, more research is needed.

## Conclusions

Some pleasing results have been published. Although more research is needed to confirm and standardize their use, several cardiopulmonary and inflammatory biomarkers have been evaluated; and some may prove useful in assessing the status of the infected animal, diagnosing pathological aspects of the disease or helping in the establishment of an accurate prognosis. Also, biomarkers have proven to be useful during the adulticide treatment and may potentially be helpful tools in the monitoring of the animals during the death of the worms.

One of the biggest drawbacks in their use is the lack of standardization that would allow a veterinary clinician to interpret values obtained from different studies; also, the low availability of some of these tests along with the high prices which, on many occasions, discourage the clinician from carrying out the tests.

D-dimer is one of the most promising of the reported biomarkers, which is particularly useful when a dog is suspected of suffering pulmonary thromboembolism. Because this is a frequent and fatal phenomenon in this disease, the use of this biomarker could help to diagnose this pathology quickly allowing an early response and treatment, substantially improving the prognosis of the affected animals.

Moreover, the preliminary results obtained with the APP, especially CRP, promise interesting results in the studies to come. The possibility of prescreening for the presence/absence of pulmonary hypertension and endothelial damage with serum analysis opens up a window of opportunities for the veterinary clinician. Furthermore, NT-proBNP and endothelin-1 are little explored but promising biomarkers. In the meantime, there are many other biomarkers to explore while new and better biomarkers continue to appear.

## Abbreviations

ANP: Atrial natriuretic peptide
APP: Acute phase proteins
CRP: C-reactive protein
DIC: Disseminated intravascular coagulation
ET-1: Endothelin-1
NT-proBNP: N-terminal pro B-type natriuretic peptide
PON-1: Paraoxonase-1
SAA: Serum amyloid A
